# *ERRF* sensitizes ERBB2-positive breast cancer cells to lapatinib treatment likely by attenuating MCL1 and ERBB2 expression

**DOI:** 10.18632/oncotarget.16425

**Published:** 2017-03-21

**Authors:** Leilei Qi, Baotong Zhang, Shiying Zhang, Xinpei Ci, Qiao Wu, Gui Ma, Ang Luo, Liya Fu, Jamie L. King, Rita Nahta, Jin-Tang Dong

**Affiliations:** ^1^ Department of Genetics and Cell Biology, College of Life Sciences, Nankai University, Tianjin 300071, China; ^2^ Emory Winship Cancer Institute, Department of Hematology and Medical Oncology, Emory University School of Medicine, Atlanta, Georgia 30322, USA; ^3^ Emory Winship Cancer Institute, Department of Pharmacology, Emory University School of Medicine, Atlanta, Georgia 30322, USA

**Keywords:** ERRF, lapatinib, ERBB2, breast cancer, drug resistance

## Abstract

Previously we found that the estrogen receptor (ER) related factor *ERRF* regulates cell proliferation and tumor growth, and its expression is positively associated with ER status and better survival but inversely associated with *ERBB2* (also named HER2) status in breast cancer. Here we report that ERRF also plays an important role in the response of ERBB2-positive breast cancer cells to lapatinib, a dual tyrosine kinase inhibitor that interrupts the ERBB2 and EGFR pathway. In ERBB2-positive breast cancer cell lines, lower levels of *ERRF* expression correlated with lapatinib resistance, restoration of *ERRF* expression in lapatinib-resistant cell lines JIMT-1 and MDA-MB-453 enhanced their lapatinib responses, and knockdown of ERRF in lapatinib sensitive cell lines BT-474 and SK-BR-3 caused lapatinib resistance. *ERRF*-enhanced lapatinib sensitivity was also confirmed in xenograft tumors of JIMT-1 cells. In patients with ERBB2-positive breast cancer, higher level of *ERRF* expression correlated with both pathologic complete response (pCR) to lapatinib and better survival. Mechanistically, *ERRF* expression in resistant cells promoted lapatinib-induced apoptosis by attenuating MCL1 and ERBB2 expression. These results suggest that *ERRF* plays an important role in lapatinib response of ERBB2-positive breast cancer, and further study of *ERRF* could lead to improved prediction and sensitivity of lapatinib response.

## INTRODUCTION

The ERBB2-positive subtype of breast cancer is characterized by gene amplification or protein overexpression of ERBB2, a member of the human epidermal growth factor receptor family. ERBB2 protein is an important marker and therapeutic target for about 30% of breast cancer patients, and such patients tend to have a shorter disease free survival and overall survival [1–6]. In addition to the ERBB2-positive subtype of breast cancer (ER and PR negative), the luminal B subtype is also ERBB2 positive, but this subtype is ER and PR positive and has different clinical characteristics and prognosis [7, 8]. For example, the luminal B subtype cancers are treated with endocrine therapies in combination with ERBB2 targeted drugs [9, 10], because the crosstalk between ER and ERBB2 can lead to endocrine therapy resistance [11–14], while ERBB2-positive subtype tumors can benefit from ERBB2 targeted drugs [15–19]. Unfortunately, patients usually develop resistance to ERBB2 target therapies within several years [19–21]. One of the targeted therapies is the Lapatinib, a dual tyrosine kinase inhibitor that interrupts the ERBB2 and EGFR pathway in the treatment of ERBB2 positive breast cancer, which is used as ditosylate and orally active [22]. Lapatinib is primarily used in patients with advanced-stage, ERBB2-positive breast cancer that has stopped responding to anthracyclines, taxanes, and herceptin [19, 23].

A number of studies have been published on the causes of lapatinib resistance, but the mechanisms are still not well understood, and effective therapies remain to be developed to overcome lapatinib resistance. For example, higher expression levels of several genes, including *ABCG2* [24], *PA2G4* [25], *BECN1* [26], *ATG5* [26], *MCL1* [27], *ER* [28], *MET* [29, 30], *SPOCK1* [31], *SRC* [32, 33], *HIF1A* [34], *DUSP2* [34], *MST1R* [35], *MAP2K1* [36], *MAP2K2* [36], *FOXM1* [36], *AXL* [37] and *YBX1* [38], have been shown to correlate with lapatinib resistance, but none of them can be used as diagnostic markers and neither have any therapeutic strategies been developed based on these molecules.

The ER related nuclear factor *ERRF* (C1orf64) was first discovered in a genome-wide sequencing study as one of the more frequently mutated genes in breast cancer [39, 40]. In a more detailed study [41], whereas the mutation of *ERRF* was not as frequent as expected, *ERRF* expression was frequently elevated in breast cancer compared to normal tissues, *ERRF* expression positively correlated with ER and PR status but negatively correlated with ERBB2 status, and knockdown of *ERRF* expression reduced tumor growth of ER and PR-positive breast cancer cells [41]. An inverse correlation between *ERRF* expression and ERBB2 status was also evident in an expression profiling study of 2000 breast cancer specimens [42]. It is thus possible that *ERRF* also plays a role in the development of ERBB2 positive breast cancer and its resistance to ERBB2-targeted therapies.

In this study, we evaluated the relationship between *ERRF* expression and the sensitivity of breast cancer cells to lapatinib in the context of ERBB2 signaling. We found that *ERRF* expression positively correlated with lapatinib sensitivity. In cultured cells, ectopic expression of *ERRF* enhanced the effect of lapatinib on cell death of JIMT-1 and MDA MB-453 cells, which expressed lower levels of *ERRF* and are resistant to lapatinib, while knockdown of *ERRF* compromised the effect of lapatinib on BT-474 and SK-BR-3 cell lines, which were sensitive to the drug and expressed higher levels of *ERRF*. The effect of *ERRF* on lapatinib was also confirmed in a xenograft model at least for the JIMT-1 cell line. We also found that *ERRF* attenuated the expression of ERBB2, which likely mediated the effect of *ERRF* on lapatinib sensitivity.

## RESULTS

### Induction of *ERRF* expression by lapatinib in lapatinib sensitive breast cancer cell lines and the correlation between *ERRF* expression and lapatinib sensitivities and better patient survival

**Figure 1:**
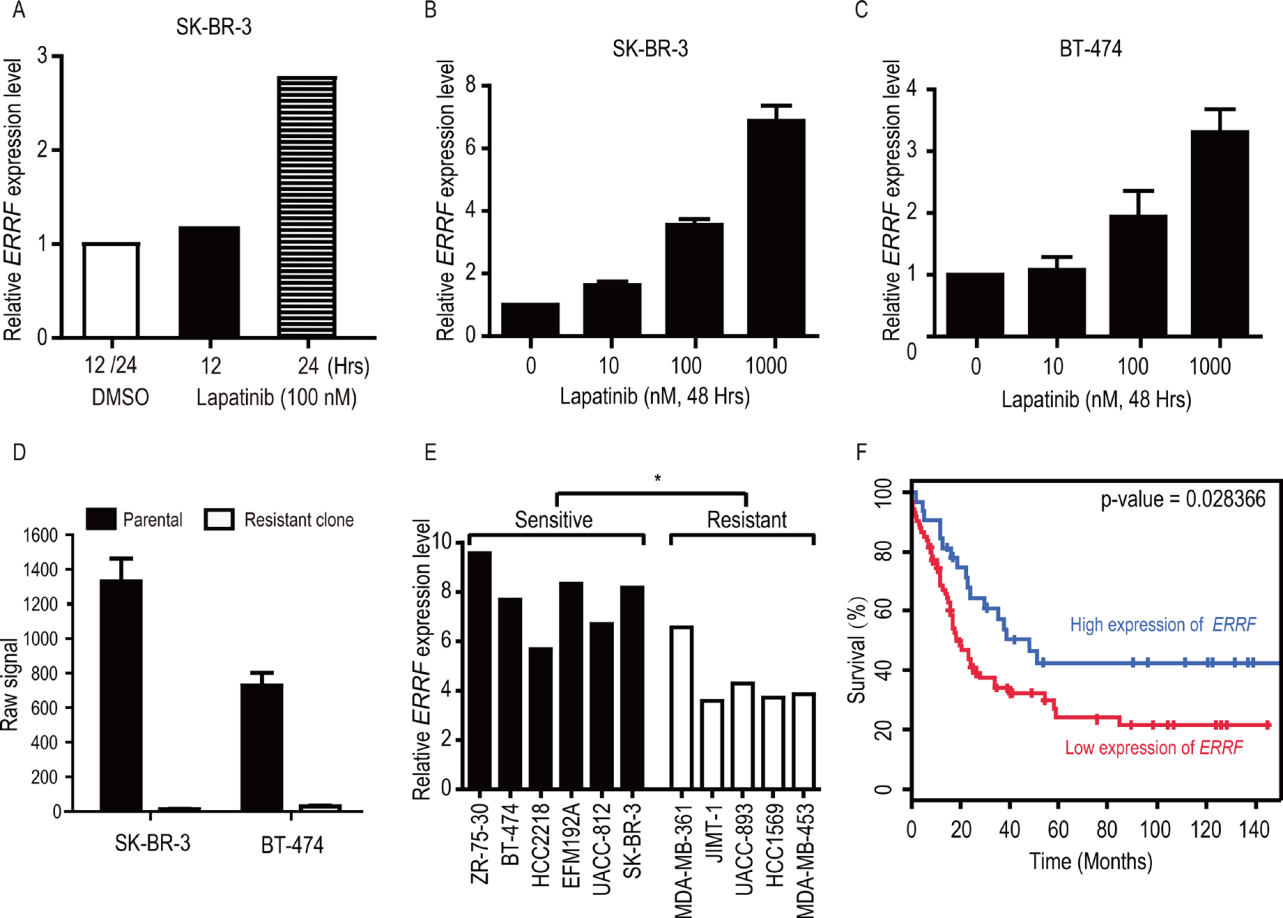
Lapatinib upregulates *ERRF* expression in SK-BR-3 and BT-474 breast cancer cell lines, and higher levels of ERRF correlate with lapatinib sensitivities and better patient survival (**A**) Illustration of *ERRF* expression after lapatinib treatment (100 nM) for 12 and 24 hours in SK-BR-3 cells based on the data from the Array Express database [43]. (**B**, **C**) Lapatinib upregulates *ERRF* expression in BT-474 and SK-BR-3 breast cancer cell lines, both express *ERRF* and respond to ERBB2 inhibition. Lapatinib treatment was at the indicated concentrations (μM) for 48 hours, and *ERRF* expression was measured by real-time RT-PCR. (**D**) Row signal of *ERRF* in SK-BR3 and BT-474 breast cancer cell lines and lapatinib resistant clones derived from them, as detected in a microarray study in the GEO database [44]. (**E**) Correlation of *ERRF* mRNA levels with sensitivities to ERBB2 inhibition in breast cancer cell lines, according to published information [45] and the CCLE database [58]. (**F**) Higher levels of *ERRF* expression are associated with better prognosis in ERBB2-positive breast cancer, as determined in the BreastMark database (HR = 0.5442, *p* = 0.028, *n* = 107).

Analysis of the Array Express database [43] showed that in the SK-BR-3 lapatinib-sensitive breast cancer cell line, treatment with lapatinib caused an upregulation in *ERRF* expression in a time-dependent manner (Figure 1A). We confirmed that lapatinib-mediated *ERRF* upregulation was also dose dependent in both SK-BR-3 and BT-474 cell lines (Figure 1B, 1C), the latter was also a lapatinib sensitive breast cancer cell line. Lapatinib resistant clones had been developed from both SK-BR-3 and BT-474 cell lines [44], and analysis of available genome-wide expression data for these resistant cells in the GEO database [44] indicates that *ERRF* mRNA expression was dramatically downregulated in the lapatinib resistant clones of SK-BR-3 and BT-474 cells (Figure 1D).

To further test the correlation between *ERRF* expression and lapatinib sensitivity, we analyzed *ERRF* expression and sensitivities of breast cancer cell lines to ERBB2 drugs including lapatinib in a published study [45] and the CCLE, and found that lapatinib sensitive cell lines expressed significantly higher levels of *ERRF* than lapatinib resistant cell lines (Figure 1E).

We also tested whether *ERRF* expression correlates with prognosis in patients with ERBB2 positive breast cancer using the BreastMark Coexpression analysis tool. Interestingly, in 107 patients with known *ERRF* expression and disease-free survival (DFS) statuses, higher levels of *ERRF* expression significantly correlated with better DFS (Figure 1F).

### Restoration of ERRF expression sensitizes lapatinib resistant ERBB2 positive breast cancer cells to lapatinib

**Figure 2:**
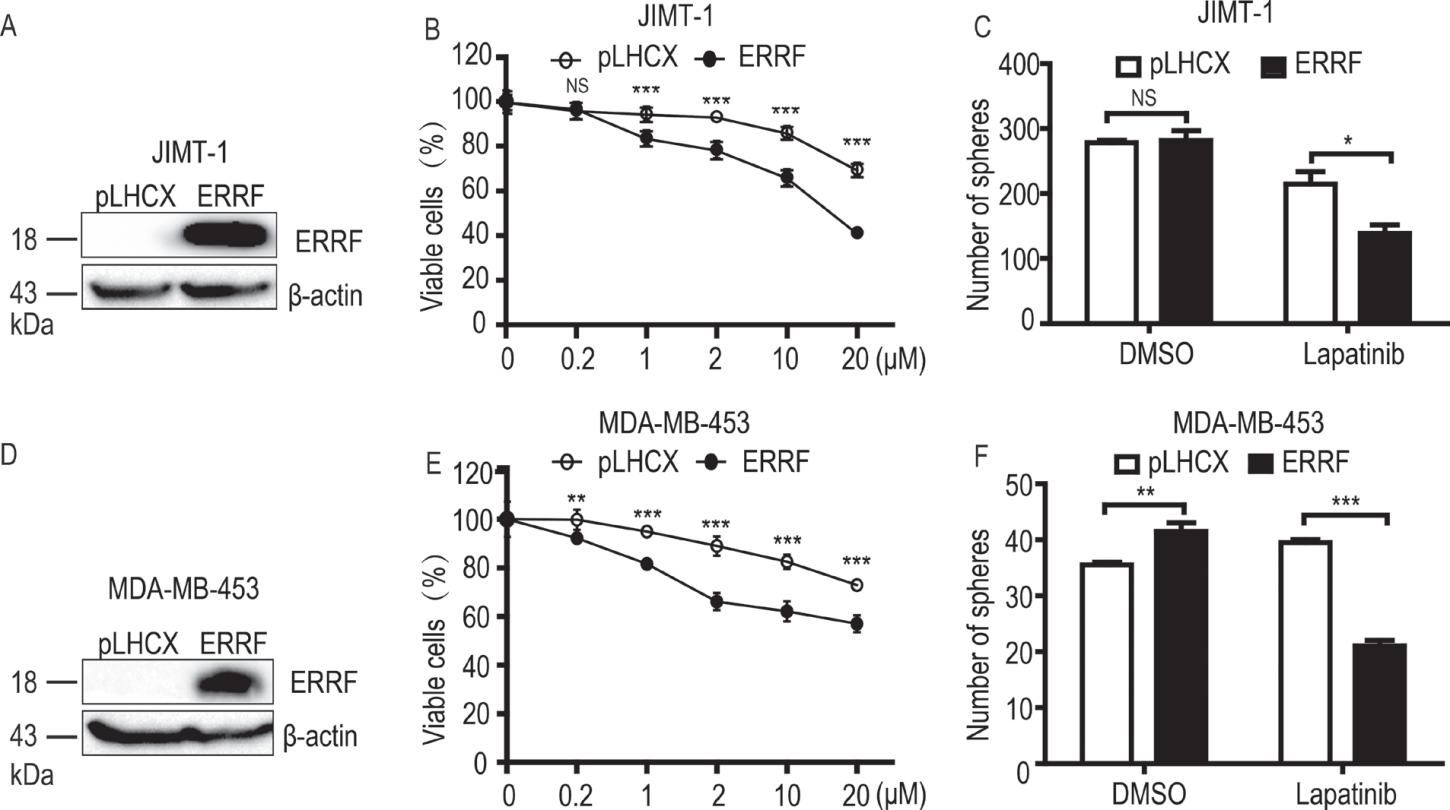
Ectopic expression of ERRF enhances lapatinib sensitivity in ERBB2-positive and lapatinib-resistant JIMT-1 and MDA-MB-453 breast cancer cell lines Lentivirus-mediated stable expression of ERRF was confirmed by western blotting (**A**, **D**) and cell viability was determined by the CCK-8 assay (**B**, **E**) and the sphere formation assay was in matrigel or in ultralow attachment plates (**C**, **F**). Lapatinib treatment was for 6 days at indicated concentrations in the CCK-8 assay and for 6 days at 2 μM in sphere formation assays. *p < 0.05; **p < 0.01; ***p < 0.001; pLHCX, vector control.

To test whether *ERRF* is functionally involved in drug sensitivity, we restored *ERRF* expression by lentiviral infection in JIMT-1, a lapatinib resistant, ERBB2 positive breast cancer cell line that expressed a lower level of *ERRF* (Figure 1E), and measured cell survival in both 2-D and 3-D cultures. Ectopic expression of *ERRF* was confirmed by western blotting (Figure 2A). Stable ERFF expression clearly enhanced the killing effect of lapatinib on JIMT-1 cells in a dose dependent manner (Figure 2B). In 3-D culture, stable ERFF expression also decreased mammosphere formation of JIMT-1 cells after lapatinib treatment, although *ERRF* had no effect on sphere formation when lapatinib was absent (Figure 2C). Consistent results were obtained in the MDA-MB-453 breast cancer cell line, which was also lapatinib resistant and ERBB2 positive and expressed a lower level of *ERRF* (Figure 2D–2F).

### Knockdown of ERRF expression desensitizes ERBB2 positive breast cancer cells to lapatinib

**Figure 3:**
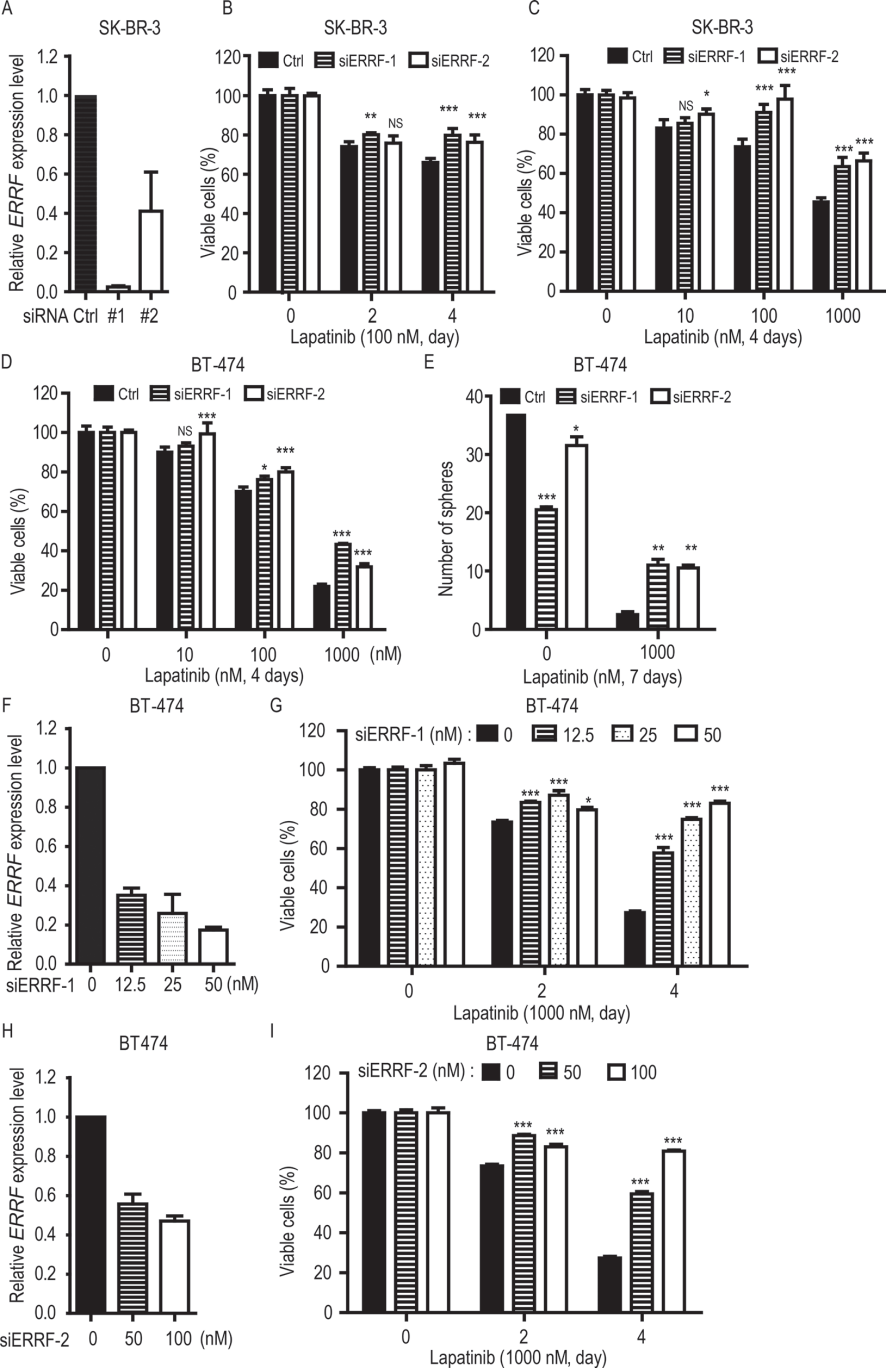
Knockdown of ERRF decreases lapatinib sensitivities in ERBB2-positive breast cancer cells SK-BR-3 (**A**–**C**) and BT-474 (**D**–**I**) cell lines were used, and the knockdown effect for siRNAs (#1 and #2) was confirmed by real-time PCR (A, F, H). The CCK-8 assay was used to detect cell viability (B, C, D, G, I), and sphere formation assay was also performed for BT-474 cells (E). Lapatinib treatments were at indicated times and doses (B, C, D). For BT-474 cells, different concentrations of siRNA were also applied (G, I) **p < 0.01; ***p < 0.001; C, negative control.

To further test the effect of *ERRF* on lapatinib sensitivity, we used two breast cancer cell lines that were ERBB2 positive and lapatinib sensitive [22, 46] and expressed higher levels of *ERRF* (Figure 1E), i.e., SK-BR-3 and BT-474. *ERRF* expression was knocked down by RNAi using two independent siRNAs [41], and the effect of knockdown was confirmed by real-time PCR (Figure 3A). CCK-8 analysis indicates that knockdown of *ERRF* expression compromised the killing effect of lapatinib on SK-BR-3 cells in a time- (Figure 3B) and dose-dependent manner (Figure 3C).

Similar results were obtained in BT-474 cells, where *ERRF* knockdown had no effect on cells treated with lower doses of lapatinib but significantly decreased drug sensitivities when higher doses of lapatinib were applied (Figure 3D). In 3-D culture, the effect was more profound, as *ERRF* knockdown increased sphere formation by several folds in lapatinib treated cells (Figure 3E). When we silenced *ERRF* by different concentrations of siRNA to simulate a range of expression levels similar to those in lapatinib resistant cell lines (Figure 3F, 3H), cell viability increased with increasing doses of *ERRF* siRNA in lapatinib treated cells in a time dependent manner (Figure 3G, 3I). The results from both SK-BR-3 and BT-474 cell lines indicate that *ERRF* downregulation causes a level of significantly reduced sensitivity to lapatinib treatment in ERBB2 positive breast cancer cells.

### ERRF expression enhances lapatinib's therapeutic effect in a preclinical model and correlates with improved clinical response to lapatinib in patients

**Figure 4:**
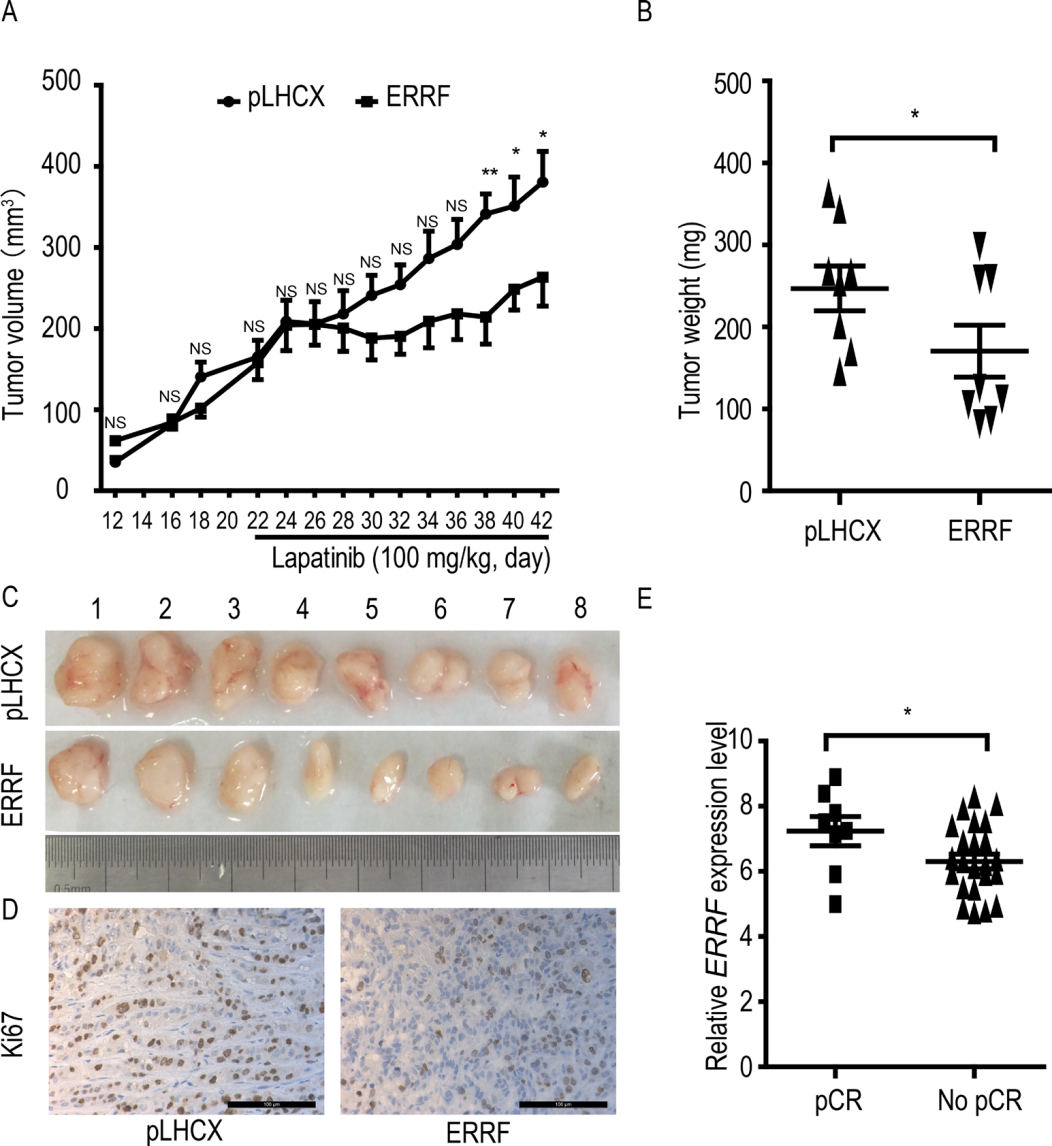
ERRF expression sensitizes xenograft breast cancer to the therapeutic effect of lapatinib and predicts response to lapatinib in breast cancer patients Lapatinib resistant and ERBB2-positive JIMT-1 cells expressing *ERRF* or vector control (pLHCX) were injected subcutaneously into nude mice, lapatinib treatment was started at day 21, and tumor volumes (**A**) weights at excision (**B**) and images (**C**) are shown. (**D**). Detection of Ki67 in tumor sections by IHC staining. (**E**) Correlation of higher levels of *ERRF* expression with pathologic complete response (pCR) in patients treated with chemotherapy combined with lapatinib, as determined using a publically available data in the GEO database. NS, not significant; *P < 0.05; **P < 0.01.

To test whether *ERRF* expression improves the therapeutic response of ERBB2 positive breast cancer to lapatinib, JIMT-1 cells stably expressing *ERRF* and the vector control were subcutaneously injected into nude mice, lapatinib treatment was applied subsequently, and tumor growth was analyzed. During the 3 weeks of tumor growth before lapatinib administration, *ERRF* expression had no detectable effect on tumor volumes (Figure 4A). After lapatinib treatment began, tumor growth in the *ERRF*-overexpressing group was arrested or significantly slowed from day 7 after lapatinib administration (Figure 4A), but tumors in the control group kept growing at the same rate before treatment. Tumor weights (Figure 4B) and tumor images (Figure 4C) at excision confirmed the effect of *ERRF* expression on the therapeutic effects of lapatinib in JIMT-1 cells. Cell proliferation rate, indicated by Ki67 IHC staining, was decreased in the group of *ERRF* overexpression under lapatinib treatment (Figure 4D).

In a phase II randomized study where drug activity and safety were evaluated in ERBB2 positive operable breast cancer, patients were treated with chemotherapy as a neoadjuvant therapy and lapatinib as a targeted therapy [47]. Analysis of available data in this study indicates that *ERRF* expression was higher in patients with pathologic complete response (pCR) than those without (Figure 4E). In this study, *ERRF* was identified in the list of 50 genes whose expression states predicted pCR in 93% of the test tumor samples [48], suggesting that patients with ERBB2 positive breast cancer, higher *ERRF* expression predicts a benefit from lapatinib treatment.

### ERRF expression enhances lapatinib-mediated apoptotic response

**Figure 5:**
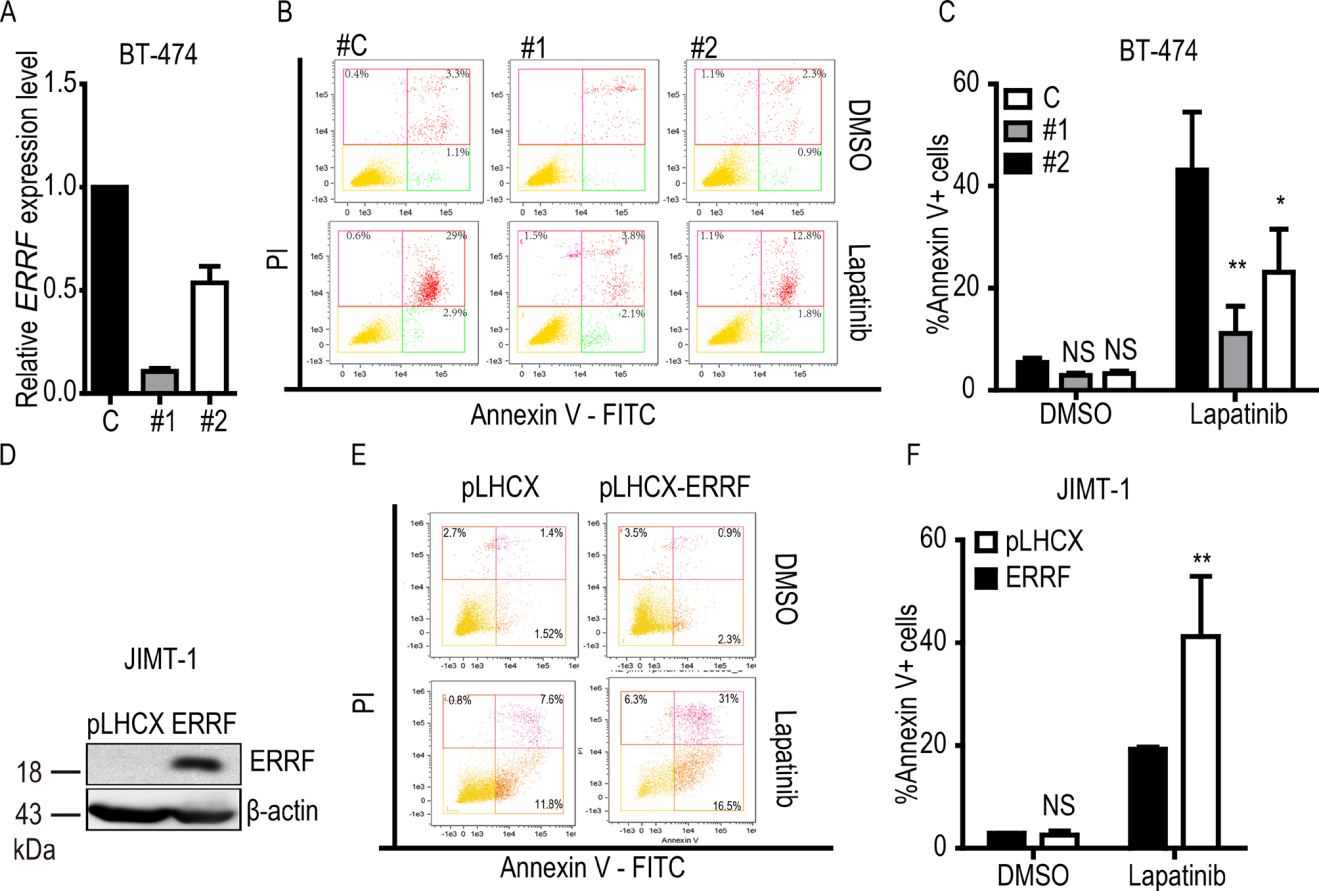
ERRF enhances lapatinib-mediated apoptosis BT-474 cells with RNAi-mediated knockdown of *ERRF* (**A**–**C**) and JIMT-1 cells overexpressing *ERRF* (**D**–**F**) were treated with 10 μM lapatinib for 24 h, stained with annexin V, and then subjected to flow cytometry to detect apoptotic cells. The knockdown of *ERRF* was confirmed by real–time PCR, while ectopic expression of ERRF was confirmed by western blotting. C, control; #1 and #2, siRNAs against ERRF. **p < 0.01; ***p < 0.001.

Lapatinib treatment induces apoptosis [49], so we examined the effect of *ERRF* on lapatinib-mediated apoptosis by flow cytometry in lapatinib sensitive BT-474 cells with the knockdown of *ERRF* (Figure 5A) and lapatinib resistant JIMT-1 cells with ectopic expression of *ERRF* (Figure 5D). Without lapatinib treatment, stable overexpression or knockdown of *ERRF* had no significant effect on apoptosis (Figure 5C, 5F). When lapatinib treatment was applied, knockdown of *ERRF* in BT-474 cells decreased the ratio of apoptotic cells (annexin V stained) from 43.2% for control siRNA to 11.1% for siRNA #1 and 23.1% for siRNA #2 (Figure 5B, 5C). In JIMT-1 cells treated with lapatinib, the vector control had 19.7% of annexin V positive cells, while cells overexpressing *ERRF* increased the ratio to 41.2% (Figure 5E, 5F). Therefore, *ERRF* expression influences the apoptotic response of ERBB2 positive breast cancer cells to lapatinib.

### ERBB2 and MCL1 appear to mediate ERRF's effect on lapatinib sensitivity

**Figure 6:**
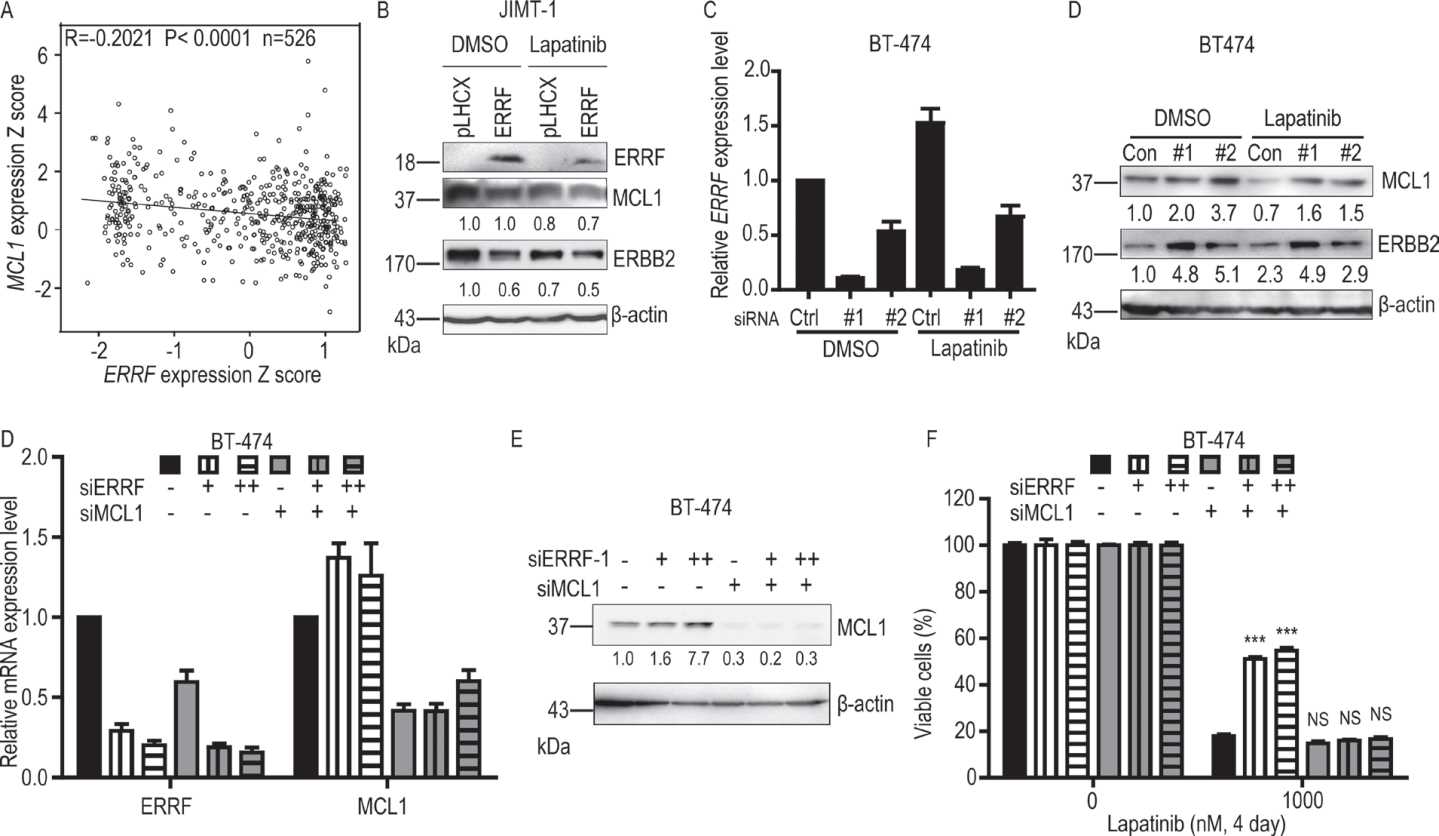
ERBB2 and MCL1 are involved in ERRF mediated sensitization to lapatinib treatment (**A**) Expression of *ERRF* inversely correlates with that of MCL1 in breast cancer samples, as determined by the Pearson correlation analysis using data from the TCGA database (Supplementary Table 1). (**B**) Overexpression of *ERRF* inhibits ERBB2 and MCL1 expression in the JIMT-1 ERBB2 positive breast cancer cell line, as determined by western blotting. (**C**, **D)**. Knockdown of *ERRF* in the BT-474 breast cancer cell line, as confirmed by real-time PCR (C), increases ERBB2 and MCL1 expression, as determined by western blotting (D). (**E**, **F)** Knockdown of *MCL1* by siRNA transfection (50 nM) rescued decreased sensitivity of BT-474 cells to lapatinib mediated by *ERRF* knockdown (12.5 nM and 25 nM siRNA). *ERRF* and MCL1 expression levels were examined by real-time PCR or western blotting. Con, control; #1 and #2, siRNAs against ERRF.

Lapatinib can inhibit tyrosine kinase activity of ERBB2/EGFR to inactivate the downstream signaling pathway. In this study, we found no correlation between *ERRF* and AKT and MAPK's phosphorylation levels (Supplementary Figure 1A). Using real-time PCR analysis, we tested the expression levels of genes that have been shown to correlate with lapatinib resistance in previous studies (Supplementary Figure 1B), and found that *MCL1* was one of the genes that were upregulated by *ERRF* knockdown. Considering that *ERRF* enhances lapatinib-induced apoptosis (Figure 5), *ERRF* expression inversely correlates with ERBB2 status [41], and *MCL1* is not only an anti-apoptotic gene that mediates lapatinib resistance in HCT116 cells [50] but also an upstream regulator of ERBB2 [51], we evaluated whether ERBB2 and MCL1 are related to *ERRF* in any way in breast cancer cells. We queried breast cancer samples in the TCGA database for those with expression information for both *ERRF* and *MCL1* (Supplementary Table 1), and found a significant inverse correlation between *ERRF* and MCL1 (Figure 6A).

We further tested whether there was a relationship between *ERRF*, MCL1, ERBB2 and lapatinib response. In JIMT-1 cells with *ERRF* overexpression, we found that treatment with 1 μM lapatinib for three days downregulated both MCL-1 and ERBB2 compared to cells without *ERRF* overexpression (Figure 6B). Consistently, silencing *ERRF* with siRNAs in BT-474 cells treated with lapatinib increased the expression of both MCL-1 and ERBB2 when compared to the negative control (Figure 6C, 6D). In BT-474 cells transfected with *ERRF* siRNA, insensitivity to lapatinib was rescued by knocking down *MCL1* (Figure 6E, 6F), further implicating MCL1 in *ERRF*-mediated lapatinib sensitivity.

## DISCUSSION

In this study, we tested whether *ERRF* plays a role in the development of resistance to ERBB2-targeted therapies in ERBB2-positive breast cancer. We evaluated the expression of *ERRF* in breast cancer in publically available databases, and correlated *ERRF* expression to responses to both ERBB2-targeted therapies and patient survival. We also tested whether *ERRF* functionally modulates the responses of ERBB2-positive breast cancer cells to the lapatinib ERBB2 inhibitor. Finally, we explored cellular and molecular mechanisms that underlie the effect of *ERRF* on lapatinib-mediated cell killing. Results from these analyses established *ERRF* as an important regulator of ERBB2 function and the effectiveness of ERBB2-targeted therapy.

*ERRF* expression predicts the sensitivity to lapatinib in ERBB2 positive breast cancer. This conclusion is supported by several lines of evidence, including the upregulation of *ERRF* by lapatinib in lapatinib-sensitive SK-BR-3 and BT-474 breast cancer cell lines (Figure 1A–1C), downregulation of *ERRF* in lapatinib-resistant clones derived from SK-BR-3 and BT-474 cells (Figure 1D) [44], and the association of *ERRF* expression with lapatinib sensitivities in breast cancer cell lines (Figure 1E) [44]. In patients with breast cancer, although no data is currently available for correlating *ERRF* expression and lapatinib sensitivities, higher levels of *ERRF* expression significantly correlated with better DFS (Figure 1F), and *ERRF* was among a panel of 50 genes predicting treatment benefit from a combination of chemotherapy and lapatinib treatment [47].

Functionally, *ERRF* indeed sensitizes ERBB2 positive breast cancer to the therapeutic effects of lapatinib. This conclusion was supported by *in vitro* and *in vivo* experiments using both lapatinib resistant and lapatinib sensitive cell lines with modulated *ERRF* expression. In both 2-D and 3-D cell culture models, expression of *ERRF* sensitizes intrinsically resistant ERBB2 positive breast cancer cells to lapatinib (Figure 2); and *in vivo* tumorigenesis assay confirmed the effect (Figure 4). On the other hand, knockdown of *ERRF* in two lapatinib-sensitive ERBB2-positive breast cancer cell lines compromised the effect of lapatinib on cell survival in 2-D and 3-D cultures (Figure 3), further indicating a necessary role of *ERRF* in breast cancer's sensitivity to lapatinib.

Mechanistically, *ERRF* promotes lapatinib-induced apoptosis likely by attenuating the expression of ERBB2 and its upstream regulator MCL-1. As expected, ectopic expression of *ERRF* increased, while knockdown of *ERRF* expression decreased, cell death induced by lapatinib in different breast cancer cell lines (Figure 5). Drug resistant cells usually have active anti-apoptotic signaling, while drug sensitive cells have more active pro-apoptotic signaling [49, 52]. MCL1 is a member of the anti-apoptotic BCL-2 family that is upregulated in tumors, and its upregulation leads to drug resistance [27, 50, 53]. MCL1 has been shown to upregulate ERBB2 expression in breast cancer cells [27]. Although the relationship between ERBB2 expression and lapatinib resistance has not been reported, mitochondrial ERBB2 (mtERBB2) appears to regulate cellular metabolism and therapeutic resistance [54]. There was a correlation between *ERRF* and *MCL1* expression in breast cancer (Figure 6A); and ectopic expression of *ERRF* decreased while the knockdown of *ERRF* increased the expression of MCL1 in breast cancer cells (Figure 6), suggesting that *ERRF* could directly downregulate MCL1 expression. Taken together with the result that knockdown of *MCL1* compromised lapatinib resistance mediated by *ERRF* knockdown (Figure 6E, 6F), it is possible that *ERRF* downregulates *MCL1* expression to promote apoptosis in breast cancer's response to lapatinib. *ERRF* expression decreased while its knockdown increased ERBB2 expression in breast cancer cells as well (Figure 6), it is also possible that *ERRF* loss upregulates ERBB2 including mtERBB2 to decrease lapatinib sensitivity. Nevertheless, how *ERRF* regulates lapatinib sensitivity in breast cancer cells largely remains unknown.

*ERRF* is a novel regulator of breast carcinogenesis involved in both the ER signaling and the ERBB2 signaling. We previously reported that *ERRF* expression positively correlates with ER and PR statuses but negatively associated with ERBB2 status [41], and knockdown of *ERRF* inhibits the proliferation and tumorigenesis of ER- and PR-positive breast cancer cells [41]. In addition, *ERRF* is transcriptionally regulated by the E2-ER signaling pathway in ER/PR-positive but ERBB2-negative breast cancer cells [55]. Our current study demonstrated that in ERBB2-positive breast cancer, *ERRF* also plays an important role, as *ERRF* sensitized such cells to lapatinib treatment, and higher levels of *ERRF* expression correlated with increased lapatinib sensitivity and better patient survival. These findings suggest that *ERRF* could be useful not only in the prediction of lapatinib responses but also in the improvement of lapatinib-based therapies of ERBB2-positive breast cancer.

## MATERIALS AND METHODS

### Cell lines and other materials

BT-474, SK-BR-3 and MDA-MB-453 breast cancer cell lines and HEK293T cells were purchased from the American Type Culture Collection (ATCC, Manassas, VA) and propagated according to ATCC's instructions. The JIMT-1 cell line was kindly provided by Dr. Rita Nahta of Emory University.

Lapatinib was purchased from LC Laboratories (Woburn, MA), hygromycin B was from Roche (Basel, Switzerland), and Matrigel was from BD Biosciences (Bedford, MA).

### Retroviral expression of ERRF

Polymerase chain reaction (PCR) was performed to amplify the coding region of *ERRF* from genomic DNA with forward primer 5′–GGAAGCTTATGGCCCCGTCAGAAG–3′ and reverse primer 5′–CCATCGATCTAATCGGCCTGCCCA–3′. PCR products were digested with Hind III and Cla I, purified, and cloned into the pLHCX vector (Clontech, Mountain View, CA). After sequencing verification, the *ERRF* plasmids or the empty vector was cotransfected with the envelope vector VSV-G and the gal/pol expression vector Ecopac (Clontech) into HEK293T cells (ATCC) using the Lipofectamine 2000 reagent (Invitrogen, Carlsbad, CA). Viruses were harvested 72 hours after transfection and filtered with 0.45 μm filters (Millipore, Billerica, MA). Cells infected with viruses were selected in medium containing hygromycin B at 200 μg/ml (Roche) for 14 days before use.

### Cytotoxicity assay

After transfecting with control siRNA or *ERRF* siRNA for 48 hours or selecting with hygromycin B for 2 weeks after viral infection, cells were dissociated with trypsin and seeded onto 96-well plates (5000 cells per well). After adhesion overnight, cells were incubated with lapatinib at a range of concentrations for 4 or 6 days. The cell counting kit-8 (CCK-8, Dojindo, Munich, Germany) was used to measure the total cell numbers. Following manufacturer instructions, 10 μL CCK-8 solution was added to each well, incubated for 1.5 hours, and, optical density (OD) was measured at the 450 nm wavelength.

### Apoptosis assay

Apoptosis was measured by staining cells with Annexin V-FITC and PI. After incubation with lapatinib for 48 hours, cells were collected, washed with cold PBS, resuspended in 100 μL of 1 × Annexin V binding buffer, stained with Annexin V and PI (BD Pharmingen) by adding 5 μL of each to a tube and incubating for 15 min in the dark at room temperature, and analyzed by flow cytometry using the FlowJo 7.6 software.

### Western blotting

Antibodies used in this study included: ERRF (1:1000 dilution, Sigma-Aldrich, St Louis, MO), ERBB2 (1:1000 dilution, OriGene, Rockville, MD), β-actin (1:5000 dilution, Sigma-Aldrich), and MCL1 (1:1000 dilution, Cell Signaling Technology, Danvers, MA). Western blotting was performed following standard protocols using WesternBright ECL (Advansta, Menlo Park, CA), and blots were photographed with the Image Quant LAS 4000 luminescent image analyzer (General Electric, Fairfield, CT). All Western blots were quantified using the ImageJ program.

### Mammosphere culture

For 2-D culture, cells were plated in ultralow attachment plates (Corning, Corning, NY) at a density of 5,000 viable cells/mL and grown in DMEM/F-12 medium supplemented with B27 (Invitrogen), 20 ng/mL EGF (Promega, WI), 20 ng/mL bFGF (BD Biosciences), and 10 μg/mL heparin (Sigma-Aldrich). For 3-D culture, cells were seeded into 8-well glass chamber slides containing 40 μL growth factor-reduced matrigel per well and cultured in the same medium as in 2-D culture. The number of spheres containing at least 15 cells was counted under a microscope following a published procedure [56].

### Tumorigenesis assay

Three to four week old female athymic BALB/c nude mice (vitalriver Beijing, China) were used. JIMT-1 cells in PBS:matrigel mixture (1:1 ratio) were injected subcutaneously into the flanks of mice at 2 × 10^5^ cells/ml and 100 μL/site. Eight mice were used in each group. When tumors reached a volume of approximately 100 mm^3^, mice were treated with either vehicle (2% DMSO with 30% PEG300 in water) or lapatinib (100 mg/kg) twice daily by oral gavage for 21 days [22]. Tumor volumes were measured every other day, and tumors were surgically isolated from mice and weighed at the end of tumorigenesis experiments.

### Immunohistochemistry

After excision, tumors were formalin-fixed, paraffin-embedded, deparaffinized and rehydrated; and tissue sections were prepared following standard procedure. Antigen retrieval was carried out by heating in sodium citrate buffer using a pressure cooker for 3 min at full pressure. Sections were incubated overnight at 4°C with Ki67 antibody (1:2000 dilution, Abcam, Cambridge, UK) and the HRP solution (Dako, Santa Clarita, CA) for 1 hour. The DAB-chromogen (Maxim, Fuzhou, China) was used for staining.

### Bioinformatics analysis

The BreastMark Breast Cancer Survival Analysis Tool (http://glados.ucd.ie/BreastMark/), an algorithm that integrates gene expression and survival data from 26 datasets on 12 different microarray platforms, was used to associate *ERRF* expression with patient survival with known ERBB2 status. The number of samples used for analysis depends on how many platforms have probes for a gene of interest and the availability of relevant clinical data [57].

We also used the Broad-Novartis Cancer Cell Line Encyclopedia (CCLE; http://www.broadinstitute.org/ccle/home) to evaluate *ERRF* expression and lapatinib sensitivity in breast cancer cell lines. Microarray or RNA-Seq-based expression data was available for all genes in this resource. We used the Gene Expression Omnibus (GEO) Database (data set GSE51889) to compare SK-BR-3 and BT-474 lapatinib resistant clones to control untreated cancer cells.

### Statistical analysis

All experimental readings were expressed as mean ± standard errors. Differences between two groups were determined by using the unpaired Student *t*-test, and *p*-values less than 0.05 were considered as statistically different.

### Novelty and impact

Development of resistance and sometimes lack of response to ERBB2-targetted therapy constitute a significant problem in the treatment of ERBB2-positive breast cancer. In this study, we found that expression of *ERRF*, an estrogen receptor related factor, predicted the sensitivity of ERBB2-positive breast cancers to lapatinib, a drug used in combination with other drugs to treat either ERBB2-positive breast cancers that have received prior therapy or metastatic, hormone receptor positive and ERBB2-postive breast cancer in postmenopausal women. Functionally, overexpression of *ERRF* sensitizes ERBB2-positive breast cancer cells to lapatinib in both *in vitro* and *in vivo* assays, and the underlying mechanism involved *ERRF*-mediated expression change of ERBB2. These findings implicate *ERRF* in the ERBB2 pathway and the response of ERBB2-positive breast cancer to lapatinib, and further studies in this area could lead to improved detection and/or treatment of ERBB2-positive breast cancer.

## SUPPLEMENTARY MATERIALS FIGURES AND TABLES




