# Supplementary material for: ERRF sensitizes ERBB2-positive breast cancer cells to lapatinib treatment likely by attenuating MCL1 and ERBB2 expression
Source: Oncotarget. 2017 Mar 21;8(22):36054–66. doi: 10.18632/oncotarget.16425 (PMC5482638; doi:10.18632/oncotarget.16425)
Supplement: Supplementary file 1 [file oncotarget-08-36054-s001.pdf]

ERRF sensitizes ERBB2-positive breast cancer cells to lapatinib treatment likely by attenuating MCL1 and ERBB2 expression

Supplementary Materials

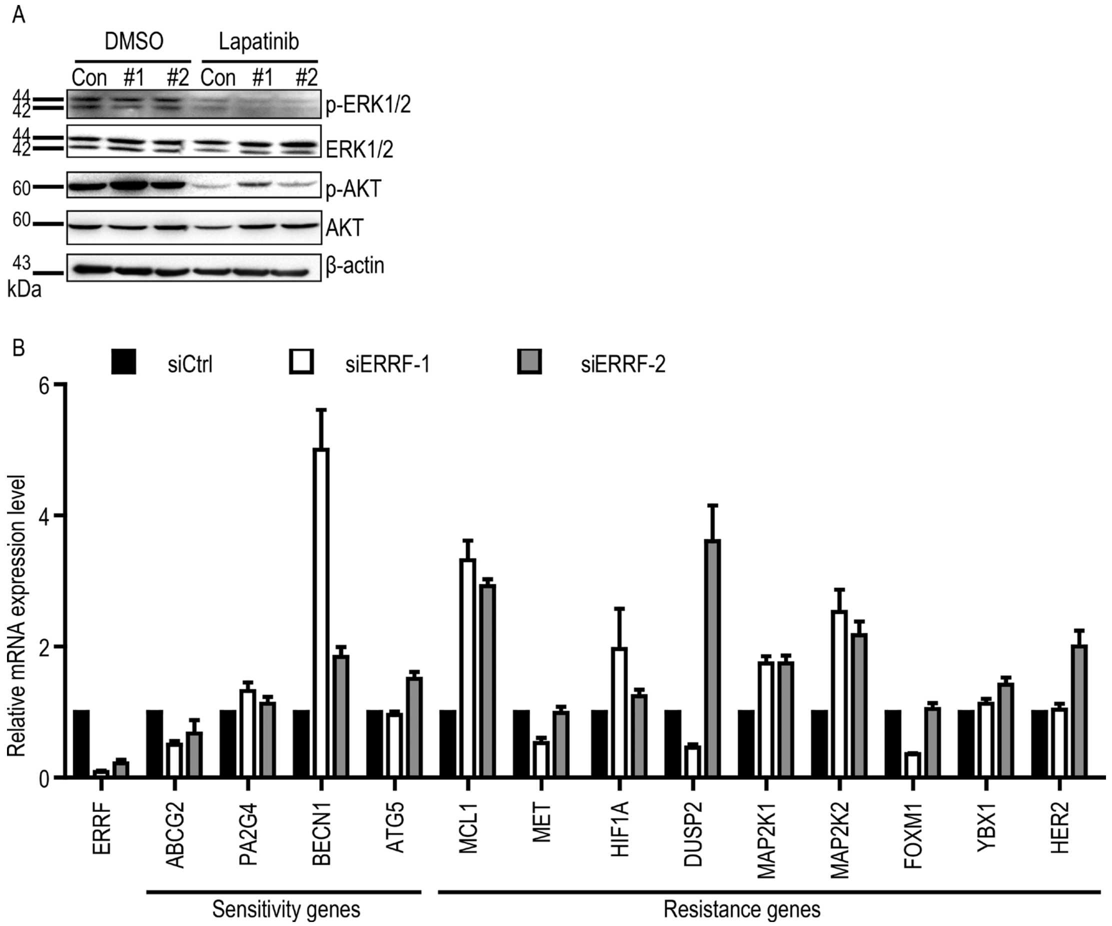

Supplementary Figure 1: Knockdown of ERRF has no obvious effect on the phosphorylation of ERK and AKT but altered the expression of multiple genes related to lapatinib sensitivity in BT-474 cells. (**A**) Cells were transfected with control siRNA or *ERRF* siRNA and then treated with lapatinib or DMSO control, and proteins were detected by western blotting. (**B**) Real-time PCR was used to detect gene expression in BT-474 cells transfected with siRNAs.

Supplementary Table 1: Invasive breast carcinoma samples in which expression levels of both ERRF and MCL1 are available in the TCGA database. See Supplementary_Table_1
